# Outcome of Occupational Latex Allergy—Work Ability and Quality of Life

**DOI:** 10.1371/journal.pone.0003459

**Published:** 2008-10-21

**Authors:** Albert Nienhaus, Kathrin Kromark, Monika Raulf-Heimsoth, Vera van Kampen, Rolf Merget

**Affiliations:** 1 Institution for Statutory Accident Insurance in the Health and Welfare Services, Hamburg, Germany; 2 BGFA-Research Institute of Occupational Medicine, German Social Accident Insurance, Ruhr University Bochum, Bochum, Germany; University of Cape Town, South Africa

## Abstract

**Objective:**

The quality of life (QOL) and work ability of health care workers allergic to natural rubber latex (NRL) were assessed after implementation of regulations on powder-free NRL gloves in Germany.

**Methods:**

196 HCW with reported NRL allergy answered a questionnaire (response rate 58%) containing the Work Ability Index (WAI), Mini Asthma Quality of Life Questionnaire (MiniAQLQ), and Dermatology Life Quality Index (DLQI).

**Results:**

63.2% still had NRL-related symptoms during the last 6 month. However on a scale from 0 to 10, the intensity of NRL-related symptoms decreased from 8.5 before to 2.3 after implementation of regulations on powder-free NRL gloves. A higher number of subjects were able to avoid NRL in the private than in the work environment (85% vs. 61%). NRL-related symptoms decreased and WAI increased with successful avoidance of NRL at workplace (b = 0.23, p = 0.003). QOL was only little affected by NRL allergy (mean: MiniAQLQ = 6.0; DLQI = 4.1).

**Conclusions:**

Although there was improvement after implementation of powder-free NRL gloves, there is still a considerable number of HCW with NRL-related symptoms. Further investigations on latex avoidance and the cause of persisiting allergic symptoms in HCW with NRL allergy are therefore needed.

## Introduction

During the 1980s, as a consequence of AIDS prophylaxis, there was a great increase in the use of powdered natural rubber latex (NRL) gloves, leading mainly in the 1990s to high numbers of work-related latex allergies [1], [2]. For Health Care Workers (HCW), a prevalence up to 17% has been described [3]–[5]. At the peak in 1998, 1,262 claims of latex allergies were filed in Germany to the Institution of Statutory Accident Insurance in the Health and Welfare Service (Berufsgenossenschaft fuer Gesundheitsdienst und Wohlfahrtspflege, BGW) [6]. After the allergen source had been identified, the use of powder-free NRL gloves was recommended and various actions were undertaken to reduce the use of powdered NRL gloves in hospitals and dental practices. In the regulatory field, a revised version of the compulsory technical regulations for dangerous substances (TRGS 540) has been enforced since 1998. This explicitly states that only low-allergen, powder-free NRL gloves are allowed at workplaces [7]. This has led to an immediate decrease in the use of powdered NRL gloves and, with a time lag of about two years, the number of latex allergies in the healthcare service has diminished too [1], [8]–[10].

Nevertheless, between 1996 and 2004, the BGW confirmed 3,490 claims of occupational latex allergies in the health care service. Most of these employees are still working in the health care service. The aim of this study was to investigate whether NRL allergy continues to influence quality of life and work ability of HCW with known NRL sensitivity.

## Methods

### Subjects

A structured self-administered questionnaire was mailed to 328 HCW from 10 zip-code areas, which were selected due to proximity to the research center. All these HCW had been reported to the BGW between 1996 and 2004 due to occupational NRL allergy. The responders gave their informed written consent to participate in the study. The study was approved by the ethic's committee of Hamburg Medical Association.

### Questionnaire

The questionnaire comprised four parts: a) demographic characteristics, occupation, allergic symptoms, b) preventive measures in the private and work environment, c) quality of life (QOL), d) work ability.

NRL-related complaints were rated on a von Korff-scale [11] from 0 to 10 for the period of most intensive complaints and the past week before the survey. The best value is 0, which means that the individual has no complaints due to NRL-allergy; 10 indicate severe complaints. The subjects were assigned to the following two categories: slight (score 0–3) and moderate/strong complaints (score 4–10). NRL avoidance was rated on a scale from 0 (NRL cannot be avoided at all) to 5 (NRL can be avoided very well). In the present study, the values 4 and 5 indicate successful NRL avoidance.

The information about quality of life covered the two weeks before the survey. For individuals with NRL-related cutaneous symptoms when filing the claim, QOL was measured with the Dermatology Life Quality Index (DLQI) [12]. The DLQI consists of 10 items concerning symptoms and feelings, daily activities, leisure, work and school, personal relationships and treatment. Each question is answered by a tick box: “not at all”, “a little”, “a lot” or “very much” and is scored from 0 to 3. The sum of the score has a range from 0 (no impairment of QOL) to 30 (maximum impairment) [12], [13].

For individuals with NRL-related respiratory symptoms when filing the claim, impairment of QOL due to asthma was measured with the Mini Asthma Quality of Life Questionnaire (MiniAQLQ) [14]. The MiniAQLQ consists of 15 items concerning symptoms, environmental stimuli, emotional function and activity limitations. The questions are scored from 1 to 7. The overall score is the mean of the responses to each of the 15 questions. The best score is 7, which means that the individual has no impairment due to asthma. 4 indicates a moderate degree of impairment and 1 severe impairment [15].

Work ability was assessed with the Work Ability Index (WAI) and was derived as the sum of seven items: current work ability compared with lifetime best, work ability in relation to the demands of job, number of current diseases diagnosed by a physician, estimated work impairment due to diseases, sick leave during the past 12 month, personal prognosis of work ability 2 years from now, mental resources. The range for the summed index is 7–49, subjects were assigned to the following three categories: poor (score 7–27), moderate (score 28–43) and good work ability (score 44–49) [16], [17]. The short version of the WAI was used [18].

### Data analysis

Categorical variables were assessed with the χ^2^-Test (Pearson). For all statistical analyses, a 0.05 level of significance was used. Odds Ratios (OR) and 95%-Confidence Intervals for NRL-related symptoms were calculated with multiple logistic regression. Modelbuilding was performed backward retraining all variables with p<0.1 in the final model. Variables associated with work ability were evaluated using multivariate linear regression analyses. All statistical calculations were performed using the SPSS 12.5 Software for Windows (SPSS Inc., Chicago, IL, USA).

## Results

The response rate was 60% (n = 196). Fourteen persons were excluded due to obviously uncertain diagnoses. The study population and the population of non-responders are described in Table 1. Non-responder analysis showed that nurses were the predominant profession, both in responders and non-responders. Older workers were more likely to respond, whereas the response rate was not influenced by gender, time period since filing the claim or the kind of work-related symptoms (cutaneous or respiratory).

**Table 1:** Characteristics of responders and non-responders.

	Responders	Non-Responders	p
	Mean (SD^1^)	Mean (SD^1^)	
Age (years)	40.2 (10.3)	37.7 (10.8)	0.020
Period between filing the claim and survey (years)	6.8 (2.4)	6.5 (2.2)	*0.552*
Work-related symptoms at the time of filing the claim	N (%)	N (%)	
cutaneous	83 (46)	63 (48)	*0.890*
respiratory	35 (19)	26 (20)	
Both	64 (35)	43 (32)	
Profession			
nurses	107 (59)	52 (39)	0.002
medical assistants	58 (32)	58 (44)	
other^2^	17 (9)	22 (17)	
Gender			
females	172 (94)	120 (91)	*0.218*
male	10 (6)	12 (9)	
Total	182 (100)	132 (100)	

1 Standard deviation.

2 Including physicians, office workers, cooks, housekeepers, caretakers, masseurs, social workers, hairdressers, beauticians, midwives.

64% of participants are still working in the same or a comparable occupation. Almost 10% of the participants gave up their profession due to NRL allergy (Table 2). Contact to NRL could be avoided by 154 subjects (85%) in the private environment and by 72 persons (61%) in the work environment. While 67 persons (37%) did not report any NRL-related symptoms during the 6 months before the survey, 41% had still itching/burning eyes, 27% shortness of breath and/or wheeze, and 24% reported contact urticaria after contact to NRL products. Work ability was good in 16% and poor in 5% of participants still working in the same or a comparable occupation; of these, 4 (out of 6) subjects could not avoid contact to NRL at the workplace (no table). The intensity of NRL-related symptoms decreased from 8.5 at the time of most intensive complaints ever to 2.3 at the time of the survey. In 92% of subjects, the most intensive complaints occurred in the year of filing the claim or earlier (no table). The mean WAI score of participants working in the same or comparable occupation was 37.8. The average MiniAQLQ score for participants with NRL-related respiratory symptoms at filing the claim was 6.0 and the mean DLQI score for participants with NRL-related cutaneous symptoms at time of filing the claim was 4.1.

**Table 2:** Occupational status, NRL avoidance, NRL-related symptoms, work ability, QOL.

Occupational status at survey	N	%
same or a comparable occupation	117	64.3
changed job profile	20	11.0
cessation of work due to NRL allergy	18	9.9
cessation of work due to other reasons	27	14.8
Successful NRL avoidance in		
private environment	154	84.6
work environment^1^	72	61.0
NRL-related symptoms during the past 6 months		
none	67	36.8
itching/reddened skin	94	51.6
urticaria	43	23.6
sneeze/runny nose	75	41.2
shortness of breath/wheeze	50	27.5
cough	39	21.4
itching or burning eyes	74	40.7
Work ability^1^		
good	19	16.3
moderate	92	78.6
poor	6	5.1
NRL-related complaints	Mean score	SD
most intensive ever	8.5	2.0
past week	2.3	2.6
WAI^1^	37.8	5.8
MiniAQLQ^2^	6.0	1.6
DLQI^3^	4.1	6.6

1 participants working in the same or a comparable occupation.

2 participants with NRL-related respiratory symptoms at the time of filing the claim.

3 participants with NRL-related cutaneous symptoms at the time of filing the claim.

The risk for NRL-related symptoms during the six month previous the survey was higher when NRL-avoidance at the workplace was incomplete (adjusted OR 3.8; 95%CI 1.62–8.89, Table 3). Incomplete private NRL-avoidance also increased the risk for NRL-related symptoms, but this effect was not statistically significant (OR 2.7; 95%CI 0.91–7.93). In those with intensive NRL-related complaints in history (above the mean of 8.3), the OR for current complaints was 3.0 (95%CI 1.55–5.88). Males were less likely than females to report recent NRL-related symptoms, but again this difference was not statistically significant (OR 0.3; 95%CI 0.07–1.28).

**Table 3:** Multivariate logistic regression analyses for factors influencing NRL-related symptoms (n = 182).

	NRL-related symptoms in last 6 month			
	yes	no		
NRL-avoidance in work environment	N (%)	N (%)*	Odds Ratio	95%CI
well/very well	44 (53.0)	39 (47.0)	1	–
: not at all-moderately well	43 (81.1)	10 (18.9)	**3.8**	**1.62–8.89**
No longer active in heath care	28 (60.9)	18 (39.1)	1,5	0.68–3.30
NRL-avoidance in private environment:				
well/very well	23 (82.1)	5 (17.9)	1	–
not at all–moderately well	92 (59.7)	62 (40.3)	2.7	0.91–7.93
Gender				
females	112 (65.5)	59 (34.5)	1	–
males	3 (27.3)	8 (72.7)	0.3	0.07–1.28
Level of most intensive complaints ever				
below mean (<8.3)	36 (47.4)	40 (52.6)	1	**–**
above mean (>8.3)	79 (74.5)	27 (25.5)	**3.0**	**1.55–5.88**

The following factors had no influence on NRL-related symptoms: NRL-related symptoms of skin versus airways at time of filing the claim, period since filing the claim to the survey, profession and age.

Subjects with NRL-related respiratory symptoms at the time of filing the claim had a lower WAI than those with cutaneous symptoms or with both types of symptoms (Table 4). The WAI increased with successful avoidance of NRL in the work environment. Cutaneous symptoms within six months before the survey were associated with a lower WAI.

**Table 4:** Multivariate linear regression analyses for factors influencing WAI (adjusted for gender and age); currently employed participants (n = 136^1^).

NRL-related symptoms at time of filing the claim	N	Median	25 (75) Percentile	standardized coefficient Beta	p
cutaneous	64	40.5	37.0 (43.0)	−0.244	0.001
respiratory	29	36.0	32.5 (41.0)		
both	43	37.0	32.0 (40.0)		
avoidance of NRL in the work environment					
not at all/occasionally/moderately well	53	38.0	34.0 (41.0)	0.226	0.003
well/very well	83	39.0	34.0 (43.0)		
NRL-related cutaneous symptoms (past 6 months)					
no	77	39.5	35.0 (44.0)	−0.243	0.002
yes	59	38.0	33.0 (41.0)		

The following factors had no influence on the WAI: level of most intensive complaints ever, level of complaints in the year of the claim, NRL-avoidance in the private environment, asthma in the 6 months before the survey, period of filing the claim to the survey.

1 differing n due to missing values.

## Discussion

While 65% of the responders reported to work in the same or a comparable occupation, only 10% abandoned their profession because of NRL allergy. Thus the present study demonstrates fewer job changes due to NRL than other studies [19]–[21]. However, the sample sizes of these studies are relatively small (up to n = 36), so that comparisons with our study are limited.

More than half the subjects continued to suffer from NRL-related symptoms but the intensity of NRL-related symptoms decreased considerably. The reason for the persistence of symptoms is presumably that only 61% of subjects reported a successful avoidance of NRL at work. Results in other studies range from similar reductions in symptoms, to greater reductions, to total elimination of symptoms, coupled to successful avoidance of NRL [22]–[25].

As the intensity of the complaints is clearly reduced, it is plausible that the influence of the continuing NRL-related symptoms on QOL and work ability is small. Thus, the average DLQI score in the present study was 4.1 on a scale from 0 to 30, which is only slightly worse than the value 3.3 found for operation theatre nurses [26]. In Finlay and Kahn's study, the average DLQI score of a control group with healthy skin was measured as 0.5 [12]. The DLQI score of 36 patients with a NRL allergy was 17.9 before diagnosis and 10.9 after diagnosis [20]. The mean DLQI scores from 200 patients with current cutaneous symptoms were 1.0 (moles), 8.9 (psoriasis) and 12.5 (atopic eczema) [12]. The mean value for all skin diseases came to 7.3. Other studies have measured mean DLQI values between 6.1 and 10.7 for patients with different skin diseases [27]–[31].

The mean MiniAQLQ score in the present study was 6.0 on a scale from 1 to 7 (7 = no impairment due to asthma), indicating that the remaining respiratory symptoms have only a slight influence on QOL. In various studies, mean MiniAQLQ scores from 4.6 to 5.4 have been found in adult asthmatics [14], [32], [33]. Twenty patients with NRL-induced occupational asthma succeeded in reducing NRL exposure. Their global AQLQ score was 5.2 [34]. Thus, the impairment in the QOL was higher than in the present study. This assessment was corroborated in a study in which the QOL was measured with another instrument than the AQLQ. The authors state that their results do not suggest a large effect on QOL from NRL-induced asthma [21]. As the evaluation of QOL is based on subjective perception of symptoms, further studies are needed to correlate the QOL with objective signs of the severity of the corresponding disease.

The mean WAI score in this study was 37.8 on a scale from 7 to 49 for persons with the same or a comparable occupation at the time of filing the claim. Only 5.1% of this group exhibited a poor work ability. In a Finnish study, 9% of the NRL-sensitised HCW belonged in the poor work ability category, 72% to the group with moderate work ability and 19% to the group with good work ability [35]. The mean WAI was given as 37.8, which is similar to the value of 39.1 found in the present study. In conclusion, the authors of the Finnish paper stated a clear association in health care workers between NRL allergy and a decrease in the WAI, which cannot be explained by age, gender, occupation, or history of atopy. This finding is corroborated by the present study.

The multivariate linear regression analyses show that successful avoidance of NRL at work leads to better WAI values. As however NRL avoidance in the private environment is more successful than at work, attention must still be paid to avoiding NRL at the workplace. This is also supported by the following finding: only six persons (5%) performing the same or comparable occupation had poor work ability. Four of these six persons had been unsuccessful in avoiding the allergen at work. In these cases, it may be assumed that improvements in the workplace would also improve work ability.

### Limitations

Because of the retrospective information on the NRL allergy, a recall bias cannot be excluded [36]. Moreover, a response shift bias is conceivable, because of the subjective perception of the symptom [37], [38], as adaptation of the internal evaluation scales could falsify the mapping of the individual quality of life. However, we think that these restrictions do not discredit the main finding of the present study, that avoidance strategies of NRL exposure has clearly reduced both the number of subjects with continuing symptoms and–more important–the intensity of symptoms.

HCWs with NRL-symptoms might be more likely to participate in the study than those with none or less NRL-related symptoms. Therefore the response rate gives rise to potential overestimation of the frequency of NRL-related symptoms. Furthermore, those who participated might tend to exaggerate their NRL-related complaints in the hope of receiving reimbursement by the insurance (BGW). We believe this potential for biases is limited, because the information obtained in the study was not given to the BGW-department which is dealing with the respective claims. Overall there are potential sources of biases that might lead to an overestimation of the prevalence and the intensity of NRL related symptoms.

However, these limitations of our data do not compromise the validity of the study conclusion: For many of the HCW with NRL allergy, the use of powder-free NRL gloves seems to be sufficient. It is not known whether symptoms in HCW with NRL allergy who use powder-free NRL gloves are indeed due to NRL. As there is still a considerable number of subjects with NRL allergy and continuing allergic symptoms further research should focus on the sources of the complaints and preventive measures.

## References

[1] Allmers H, Schmengler J, Skudlik C (2002). Primary prevention of natural rubber latex allergy in the German health care system through education and intervention.. J Allergy Clin Immunol.

[2] Groneberg DA, Nowak D, Wussow A (2006). Fischer A. Chronic cough due to occupational factors.. J Occup Med Toxicol.

[3] Yassin MS, Lierl MB, Fischer TJ, O'Brien K, Cross J (1994). Latex allergy in hospital employees.. Ann Allergy.

[4] Arellano R, Bradley J, Sussman G (1992). Prevalence of latex sensitization among hospital physicians occupationally exposed to latex gloves.. Anesthesiology.

[5] Horwitz IB, Arvey RD (2000). Workers' compensation claims from latex glove use: a longitudinal analysis of Minnesota data from 1988 to 1997.. J Occup Environ Med.

[6] Latza U, Haamann F, Baur X (2005). Effectiveness of a nationwide interdisciplinary preventive programme for latex allergy.. Int Arch Occup Environ Health.

[7] Allmers H, Schmengler J, John SM (2004). Decreasing incidence of occupational contact urticaria caused by natural rubber latex allergy in German health care workers.. J Allergy Clin Immunol.

[8] Raulf-Heimsoth M, Sander I, Rihs HP, Merget R, Brüning T (2003). Latex Allergy: State of the Art.. Akt Dermatol.

[9] Rueff F, Schopf P, Putz K, Przybilla B (2004). Effect of reduced exposure on natural rubber latex sensitization in health care workers.. Ann Allergy Asthma Immunol.

[10] Filon FL, Radman G (2006). Latex allergy: a follow up study of 1040 healthcare workers.. Occup Environ Med.

[11] Von Korff M, Ormel J, Keefe FJ, Dworkin SF (1992). Grading the severity of chronic pain.. Pain.

[12] Finlay AY, Khan GK (1994). Dermatology Life Quality Index (DLQI)–a simple practical measure for routine clinical use.. Clin Exp Dermatol.

[13] Lewis V, Finlay AY (2004). 10 years experience of the Dermatology Life Quality Index (DLQI).. J Investig Dermatol Symp Proc.

[14] Juniper EF, Guyatt GH, Cox FM, Ferrie PJ, King DR (1999). Development and validation of the Mini Asthma Quality of Life Questionnaire.. Eur Respir J.

[15] Juniper EF, Guyatt GH, Epstein RS, Ferrie PJ, Jaeschke R (1992). Evaluation of impairment of health related quality of life in asthma: development of a questionnaire for use in clinical trials.. Thorax.

[16] Tuomi K, Ilmarinen J, Eskelinen L, Jarvinen E, Toikkanen J (1991). Prevalence and incidence rates of diseases and work ability in different work categories of municipal occupations.. Scand J Work Environ Health.

[17] Ilmarinen J, Tuomi K, Klockars M (1997). Changes in the work ability of active employees over an 11-year period.. Scand J Work Environ Health.

[18] Thinschmidt M, Seibt R (2007). Work-Ability-Index–Comparison of the long and short version of illness diagnoses by means of a German random sample.. Zbl Arbeitsmed.

[19] Acero S, Alvarez MJ, Garcia BE, Echechipia S, Olaguibel JM (2003). Occupational asthma from natural rubber latex. Specific inhalation challenge test and evolution.. J Investig Allergol Clin Immunol.

[20] Lewis VJ, Chowdhury MM, Statham BN (2004). Natural rubber latex allergy: the impact on lifestyle and quality of life.. Contact Derm.

[21] Al-Otaibi S, Tarlo SM, House R (2005). Quality of life in patients with latex allergy.. Occup Med (Lond).

[22] Turjanmaa K, Kanto M, Kautiainen H, Reunala T, Palosuo T (2002). Long-term outcome of 160 adult patients with natural rubber latex allergy.. J Allergy Clin Immunol.

[23] Korniewicz DM, Chookaew N, El-Masri M, Mudd K, Bollinger ME (2005). Conversion to low-protein, powder-free surgical gloves: is it worth the cost?. AAOHN J.

[24] Hemery ML, Dhivert-Donnadieu H, Verdier R, Dujols P, Godard P (2004). A clinical and serological follow-up study of health care workers allergic to natural rubber latex who had followed a latex avoidance programme.. Allergy.

[25] Edelstam G, Arvanius L, Karlsson G (2002). Glove powder in the hospital environment–consequences for healthcare workers.. Int Arch Occup Environ Health.

[26] Hachem JP, De Paepe K, Sterckx G, Kaufman L, Rogiers V (2002). Evaluation of biophysical and clinical parameters of skin barrier function among hospital workers.. Contact Derm.

[27] Gradwell C, Thomas KS, English JS, Williams HC (2002). A randomized controlled trial of nurse follow-up clinics: do they help patients and do they free up consultants' time?. Br J Dermatol.

[28] Hutchings CV, Shum KW, Gawkrodger DJ (2001). Occupational contact dermatitis has an appreciable impact on quality of life.. Contact Derm.

[29] Kernick D, Cox A, Powell R, Reinhold D, Sawkins J (2000). A cost consequence study of the impact of a dermatology-trained practice nurse on the quality of life of primary care patients with eczema and psoriasis.. Br J Gen Pract.

[30] Reilly MC, Lavin PT, Kahler KH, Pariser DM (2003). Validation of the Dermatology Life Quality Index and the Work Productivity and Activity Impairment-Chronic Hand Dermatitis questionnaire in chronic hand dermatitis.. J Am Acad Dermatol.

[31] Hahn HB, Melfi CA, Chuang TY, Lewis CW, Gonin R (2001). Use of the Dermatology Life Quality Index (DLQI) in a midwestern US urban clinic.. J Am Acad Dermatol.

[32] Ind PW, Haughney J, Price D, Rosen JP, Kennelly J (2004). Adjustable and fixed dosing with budesonide/ formoterol via a single inhaler in asthma patients: the ASSURE study.. Respir Med.

[33] Pinnock H, Juniper EF, Sheikh A (2005). Concordance between supervised and postal administration of the Mini Asthma Quality of Life Questionnaire (MiniAQLQ) and Asthma Control Questionnaire (ACQ) was very high.. J Clin Epidemiol.

[34] Vandenplas O, Jamart J, Delwiche JP, Evrard G, Larbanois A (2002). Occupational asthma caused by natural rubber latex: outcome according to cessation or reduction of exposure.. J Allergy Clin Immunol.

[35] Kujala VM, Karvonen J, Laara E, Kanerva L, Estlander T (1997). Postal questionnaire study of disability associated with latex allergy among health care workers in Finland.. Am J Ind Med.

[36] Hennekens CH, Buring JE (1987). Epidemiology in Medicine.

[37] Schwartz CE, Sprangers MA (1999). Methodological approaches for assessing response shift in longitudinal health-related quality-of-life research.. Soc Sci Med.

[38] Schwartz CE, Bode R, Repucci N, Becker J, Sprangers MA (2006). The clinical significance of adaptation to changing health: a meta-analysis of response shift.. Qual Life Res.

